# When Falls in Alcohol Detox Are Not Wernicke’s: Diagnostic Overshadowing of a Right Thalamic Stroke

**DOI:** 10.1192/bjo.2026.11904

**Published:** 2026-06-30

**Authors:** Oriyomi Shittu, Abhi Salvaji

**Affiliations:** Rotherham Doncaster and South Humber NHS Foundation Trust, Doncaster, United Kingdom

## Abstract

**Aims:**

**BACKGROUND**

Neurological deterioration during alcohol detoxification is commonly attributed to alcohol-related complications, particularly Wernicke’s encephalopathy, withdrawal syndromes or side effect of detox treatment.While such diagnostic pathways are clinically appropriate, they may predispose to premature diagnostic closure. This case highlights the risk of diagnostic overshadowing when alternative neurovascular pathology presents with overlapping features.

**AIMS**

1. To examine diagnostic challenges associated with recurrent falls and neurological symptoms during inpatient alcohol detoxification

2. Highlight the clinical overlap between Wernicke’s encephalopathy and thalamic stroke, necessitating early neuroimaging

3. Emphasise the importance of structured reassessment and vigilance during week-two detox complications.

**Methods:**

A retrospective single-case clinical analysis was undertaken within an inpatient addiction detoxification unit. Clinical records, nursing observations, withdrawal monitoring tools, medical reviews, and neuroimaging reports were examined to reconstruct the diagnostic trajectory, differential formulation, and escalation decisions.

**Case Presentation:**

A 60-year-old man was admitted for planned alcohol detoxification, reporting daily consumption of approximately 14 units of lager with a Severity of Alcohol Dependence Questionnaire (SADQ) score of 28. He had no prior detoxifications, no history of delirium tremens, and no active psychiatric comorbidity. Past medical history included thrombocytopenia and abnormal liver function tests attributed to non-alcoholic fatty liver disease. Baseline physical examination was unremarkable.

During week two of detoxification, he developed recurrent falls preceded by dizziness and a “funny head” sensation. One episode occurred after rising from bed; another followed standing from the toilet. There was no loss of consciousness, seizure activity, or incontinence. Subsequent review identified a new broad-based gait,nystagmus, left upper limb hypertonia, and subtle postural weakness, although other cranial nerve examination and speech remained intact. Cognitive biases (anchoring to withdrawal, premature closure, and diagnostic overshadowing) were identified as potential contributors to delayed escalation. Initial differentials included Wernicke’s encephalopathy, hypotensive episodes related to antihypertensive use, high dose in detox reducing regime and transient ischaemic attack. The patient initially declined emergency transfer due to lack of confidence in emergency department waiting times, but was urgently escalated for acute hospital assessment and a brain scan following clinical concern.

**Results:**

Emergency department evaluation noted nystagmus, gait ataxia, and memory impairment. MRI revealed an**acute right thalamic infarct**, with additional cerebellar microhaemorrhages and chronic small-vessel disease. The thalamic lesion explained the patient’s gait disturbance, postural instability, and subtle motor changes, which had initially mimicked Wernicke’s encephalopathy.

Outcome:

The patient was transferred to neurology, commenced on stroke-appropriate management, and referred for physiotherapy. He continued relapse-prevention work with our community alcohol services and was started on acamprosate. This case demonstrates how acute stroke can present with symptoms easily misattributed to alcohol withdrawal syndromes, underscoring the need for repeated assessment and a low threshold for neuroimaging when the clinical picture evolves.

**Conclusion:**

This case underscores the potential for neurovascular events to mimic alcohol-related neurological syndromes during detoxification. Thalamic infarcts may present with gait disturbance, cognitive change, and ocular signs that overlap with Wernicke’s encephalopathy. Clinicians should maintain a broad differential when new focal signs, persistent/new ataxia, or incomplete response to thiamine therapy emerge. Early neuroimaging is essential to avoid diagnostic delay and optimise neurological outcomes.

